# Workload and Mental Well-Being of Homeworkers: The Mediating Effects of Work-Family Conflict, Sleeping Problems, and Work Engagement

**DOI:** 10.1097/JOM.0000000000002659

**Published:** 2022-08-08

**Authors:** Salvatore Zappalà, Erasmus Keli Swanzy, Ferdinando Toscano

**Affiliations:** From the Department of Psychology, Alma Mater Studiorum–University of Bologna, Cesena, Italy.

**Keywords:** homework, workload, work-family conflict, sleeping problems, work engagement, mental well-being

## Abstract

Based on the Conservation of Resources theory, this cross-sectional study investigates the relationship between workload experienced by employees when working at home and their mental well-being. Work-family conflict, sleeping problems, and work engagement are proposed as mediators.

**Methods:**

A sample of 11,501 homeworkers was drawn from the sixth wave of the European Working Condition Survey data set.

**Results:**

Unlike the expected, the higher the workload, the higher the mental well-being of employees. However, as expected, high workload was correlated with lower well-being when indirect effects through work-family conflict, sleep problems, and work engagement were considered. Similarly, the total effect of workload on mental well-being was negative.

**Conclusions:**

The study suggests that organizations should pay more attention to the amount of workload experienced by their homeworkers because it may be harmful to their health and well-being.

## Abstract 2

In a non-pandemic setting, this study in homeworkers helps to identify the mechanisms by which employees' workload affects their mental well-being. The results show that work-family conflict, sleeping problems, and work engagement are key variables that make the effects of workload involved in reducing the homeworkers' well-being.

The percentage of employees working at home has risen over recent decades.^1^ This way of working is called homeworking or, sometimes with slight conceptual differences, home-based teleworking. For reasons related to the COVID-19 emergency, it has been exponentially adopted in many organizations.

Scientific literature has identified several advantages of homeworking, such as homeworkers’ greater autonomy, increased job satisfaction and flexibility to deal with work-family demands, and limited traveling and time and cost savings for both organizations and workers.^2^ However, in addition to benefits, literature identified social isolation, technostress, or workaholism as potential drawbacks of homework.^3–7^ These contrasting results about homework lead to no consensus as to whether homeworking is good or bad for homeworkers.^2,3,8,9^

A particular concern about homework is employees’ mental well-being. Recent research suggests that working from home may affect mental well-being because this work arrangement increases work/family conflicts and employees’ feelings of loneliness.^10,11^ Furthermore, recent studies found that working from home leads to working at higher speed, meeting tight deadlines, greater work intensification, and overworking, which affect employees’ mental well-being.^12–14^ Accordingly, in this study, we explore if workload is related to homeworkers’ mental well-being.

Research investigating how workload influences the well-being of employees is still scarce and scant^15,16^; even more limited is the literature on the effects of workload on the mental well-being of homeworkers.^11,12,17^ However, recent studies conducted during the COVID-19 pandemic observed that home workers’ workload negatively influenced their well-being by increasing their work-family conflict.^11^

We investigated the relationship between homeworkers’ workload and well-being for three reasons. First, we believe it is essential to explore the relationship between workload and well-being because work conditions for homework are different from work conditions experienced at the office. For instance, homeworkers may experience more intrusions from family domains during homeworking.^18^ A high workload may affect homeworkers differently than office workers and employees working remotely in other locations than the home. Second, considering the increase in homeworkers during the COVID-19 pandemic and that organizations were not prepared to implement homeworking for many or most of their workforce,^19^ it is crucial to explore how workload is related to homeworkers’ well-being, to assist organizations in allocating reasonable workload to homeworkers. Third, the inconsistencies about the benefits of homeworking suggest that understanding how to enhance homeworkers’ well-being considering their workload may be a valuable research avenue.

We examined the relationship between homeworkers’ workload and their well-being by investigating multiple mediators that may influence this relationship. Thus, we based our argument on the Conservation of Resources (COR) theory^20^ to explain how homeworkers’ workload may significantly influence their well-being by focusing on three potential mediating variables: work-family conflict, sleeping problems, and work engagement.

## BACKGROUND

### Workload and Mental Well-being

Workload is the intensity or the extent of work assigned to an employee in a specific time frame.^21^ Based on this definition, homeworkers’ workload can be explained as the intensity or amount of job tasks accomplished within a specific time frame during homeworking.

The COR model posits that individuals endeavor to acquire, keep, foster, and guard things that they value (such as health, well-being, and family, but also objects, such as cars or tools for work, or energy resources, such as money or knowledge) and that well-being is at risk when people perceive the threat or the actual loss of one resource.^20,22^ According to this theory, when employees perceive or experience an increased workload, they have to use resources (eg, time and energy) to cope with it. This may result in the depletion and loss of those same resources that could have been devoted to personal commitments and social connections. This awareness causes homeworkers to experience stress, negatively affecting their mental well-being.^22^

Different studies reported that workload negatively affects employees’ mental well-being, supporting the assertion made by the COR theory. For example, in a traditional work context, Aalto et al^23^ conducted a study on more than 1000 physicians and found that workload was negatively associated with physicians’ mental well-being. Angioha et al^24^ observed that workload significantly and negatively affected the mental well-being of 650 government workers. Other studies supported the assertion that employees’ workload negatively affects their mental well-being.^25–27^ We argue that the same process is also valid for homeworkers since previous studies^12–14^ found that homeworkers are exposed to higher work intensification, work at high speed to meet tight deadlines, and overwork during a limited remote work time. Therefore, based on COR theory and the review of literature, we posit that:

**H1**: Workload experienced by homeworkers is negatively related to their mental well-being.

### Workload, Work-Family Conflict, and Mental Well-being

Work-family conflict is a topic widely explored in organizational literature because of its impact on individual and organizational outcomes.^28^ It expresses the role conflict occurring because of incompatible demands between work and family domains.^29^ Prior research has shown that the work-family conflict experienced by employees is significantly predicted by workload,^30^ a result in line with the COR theory. In fact, the COR theory posits that people strive to obtain and conserve essential resources for social bonds such as family and friends.^20,22^ Therefore, increased workload implies that individuals have to decrease the time and energy devoted to family members and family needs to meet the increased workload. Spending more time working because of a higher workload may often leave homeworkers emotionally exhausted, physically drained, and unable to have time and energy for family activities.^31^ Faced with increased time and energy devoted to work rather than family, homeworkers may struggle to meet family needs, leading to work-family conflict.

In turn, work-family conflict may negatively affect employees’ work engagement.^28,32^ A high work-family conflict requires resources to manage it, leaving workers with fewer resources to invest and diminishing employees’ work engagement. Obrenovic et al^33^ explained that work-family conflict diminishes employees’ mental resources, affecting work engagement. Other studies indicated that work-family conflict experienced by workers negatively and significantly affects their work engagement.^32,34^ In light of these empirical findings, we extend these results to homeworkers and, therefore, expect that their work-family conflict may negatively affect their work engagement.

The second corollary of the COR theory provides key cues to understand better the relationship between workload, work-family conflict, and well-being. This corollary emphasizes the spiral nature of resource loss and suggests that the initial loss of resources threatens the conservation of the remaining resources.^22^ Hobfoll et al^22^ explain that “because resource loss is more powerful than resource gain, and because stress occurs when resources are lost, individuals and organizations have fewer resources to offset resource loss at each iteration of the stress spiral. This creates resource loss spirals whereby losses gain in both impact and momentum” (p 107). Therefore, the initial loss of time and energy resources because of a higher workload threats the possibility to use the remaining resources, such as those related to relationships with family members. The actual loss of resources due to higher workload and the perceived threat of losing another resource, in this case, the family support resulting in work-family conflict, may gain both impact and momentum and further threaten other resources (eg, health and well-being), generating a spillover effect or what Hobfoll calls “spiral loss.” Building on the spiral loss of resources of the COR theory, we expect that the workload experienced by homeworkers is positively related to employees’ work-family conflicts, which in turn is negatively related to mental well-being. Therefore, we propose the following hypotheses:

**H2a**: Workload experienced by homeworkers is positively related to work-family conflict.
**H2b**: Homeworkers’ work-family conflict is negatively related to work engagement.
**H2c**: Homeworkers’ work-family conflict is negatively related to mental well-being.
**H2d**: The negative relationship between workload experienced by homeworkers and mental well-being is mediated by work-family conflict.

### Workload, Sleeping Problems, and Mental Well-being

According to the empirical study by Aalto et al,^23^ an increase in workload may negatively affect employees’ quality of sleep, leading to sleeping problems. Similar results also emerged from the research by Huyghebaert et al,^15^ who found that increased workload might lead to impaired sleep quality and consequent emotional exhaustion. A meta-analysis of 79 studies conducted by Nixon et al^35^ found that employees reporting higher workload reported sleeping problems due to the stress and exhaustion accompanying high workload. Based on this literature, we propose extending these findings to homeworkers by posing that their workload is significantly and positively related to their sleeping problems.

Sleeping problems are related to decreased work engagement.^36^ According to Barber et al,^36^ this occurs because a good sleep quality helps replenish and enhance self-regulatory resources after being exhausted or drained. On the contrary, sleeping problems may hinder a person from restocking self-regulatory resources depleted throughout the day. Accordingly, COR theory's desperation principle argues that people enter into a defensive mode to conserve remaining resources when previous ones have been stretched and drained.^22^ This implies that employees would be less inclined to invest more resources into the tasks they have to accomplish when their self-regulatory resources have not been fully replenished due to sleeping problems.^37^ Hence, it is possible to expect that homeworkers’ sleeping problems may harm their work engagement.

Prior studies found a relationship between sleeping problems and employees’ mental well-being.^38,39^ The rationale of this result is that sleep is crucial in the optimum physiological and human psychological functioning,^36^ and individuals who experience sleeping problems have poorer mental well-being than individuals not having such problems.^40^ In fact, sleeping problems influence people's moods and emotions, leading to anxiety and depression.^40,41^ This scenario is fully compatible with the spiral loss of resources in the COR theory. Hence, we expect that sleeping problems experienced by homeworkers because of increased workloads would have a significant adverse effect on their mental well-being. In particular, we believe that homeworkers’ workload may result in sleeping problems, which, in turn, decrease mental well-being. Thus, we posit that

**H3a**: Workload experienced by homeworkers is positively related to sleeping problems.
**H3b**: Sleeping problems experienced by homeworkers are negatively related to work engagement.
**H3c**: Sleeping problems experienced by homeworkers are negatively related to mental well-being.
**H3d**: Homeworkers’ workload has a negative indirect effect on well-being via the mediation of sleeping problems.

### Workload, Work Engagement, and Mental Well-Being

Work engagement is defined as “a positive, fulfilling, work-related state of mind characterized by vigor, dedication, and absorption”^42^(p 74). Empirical findings show that workload decreases employees’ work engagement.^43–45^ At the same time, the desperation principle of COR theory states that people get into a state of defensive mode to preserve resources when previous resources have been stretched and drained.^22^ According to this rationale, workers would be less inclined to invest more resources into their work tasks when they feel too exhausted or physically drained due to the high workload. Hence, even homeworkers who experience the loss of resources such as time and energy due to increased workload may not be able to invest more time and energy into their work tasks, thereby negatively affecting their work engagement. Therefore, we propose that homeworkers’ workload negatively affects work engagement.

Regarding the effects of work engagement on the mental well-being of employees, Radic et al^46^ suggested that more studies should examine this relationship. However, the existing research on work engagement and mental well-being found, in general, a positive relationship between these two constructs.^47–49^ Yang et al^50^ argue that work engagement is among the most significant drivers of job performance and the effort employees put into their work, thus increasing mental well-being. Therefore, work engagement should, in turn, contribute to self-development, leading to increased mental well-being. This expectation is in line with COR theory and, in particular, its second and third corollaries about resource loss cycles and gains spirals. Considering work engagement as a motivational resource, from which to obtain energy and dedication to important activities for individuals,^42^ in the gain spiral, an increase in work engagement should lead to an increase in personal well-being, and likewise, a loss of engagement should worsen employees’ well-being. Based on the reviewed literature, we suggest that homeworkers’ workload is negatively related to work engagement, which, in turn, is positively related to mental well-being. Hence, we propose the following hypotheses:

**H4a**: Workload experienced by homeworkers is negatively related to work engagement.
**H4b**: Homeworkers’ work engagement is positively related to mental well-being.
**H4c**: There is a negative indirect effect of homeworkers’ workload on mental well-being via work engagement.

**FIGURE 1:**
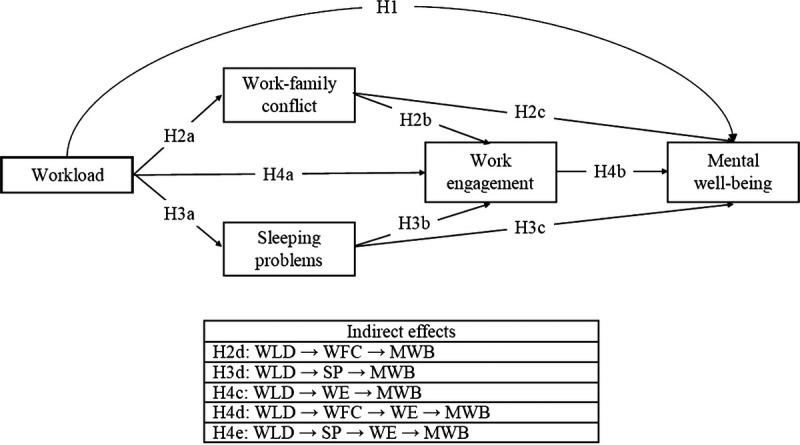
Research model for the study.

Finally, considering the mediation effect of work engagement between workload and mental well-being, the direct effect of workload on work-family conflict (H2a) and sleeping problems (H3a), and also the direct effect of work-family conflict (H2b) and sleeping problems (H3b) on work engagement, we posit two sequential mediation effects:

**H4d**: There is a negative indirect effect of homeworkers’ workload on mental well-being via work-family conflict and work engagement.
**H4e**: There is a negative indirect effect of homeworkers’ workload on mental well-being via sleeping problems and work engagement (Fig. 1).

## METHODOLOGY

### Data Sources

The present study used data from the European Working Condition Survey (EWCS) conducted every 5 years, since 1990, by the European Foundation for the Improvement of Living and Working Conditions.^51^ The EWCS is a large-scale survey that provides cross-sectional data using random samples of workers in European countries, focusing on their work-life balance, working conditions, health, employment conditions, working environments, and well-being.^52^ The Eurofound is a European Union body established by the European Council to offer better information and expert counsel on workers’ living conditions, changes in industrial relations and management among European countries, and contribute to the design and improvement of working and living conditions of workers in Europe.^52^ Researchers have highly recognized the quality of the EWCS data set.^53,54^

### Sample

We used data of the sixth wave of EWCS collected in 2015, the most recently available data set as of the writing of this contribution.^51^ The sampling procedure used for the survey was a multistage and stratified random sampling where each country was stratified into strata based on the geographical region and the level of urbanization. For our study, we extracted from the data set only respondents who reported having worked at home, answering to the following item: “How often have you worked in each location during the last 12 months—Your own home?” Participants that selected “never” were excluded from the study, whereas participants who selected “less often” to “daily” were included in the study. As a result, we obtained a sample of 11,501 homeworkers from 35 different countries.

### Measures

The scales of the Eurofound survey used in this study are reported below. For all the scales, we reversed the data so that the higher the score, the higher the presence of the variable.

Workload: Two items, on a Likert scale from 1 (never) to 7 (all of the time), were used to measure homeworkers’ workload. The two items are as follows: “Does your job involve working to tight deadlines?” and “Does your job involve working at very high speed?”

Work-family conflict: Work-family conflict was measured using three items on a 5-point Likert scale from 1 (never) to 5 (always). The items are as follows: “How often have you… 1) kept worrying about work when you were not working? 2) felt too tired after work to do some of the household jobs which need to be done? and 3) found that your job prevented you from giving the time you wanted to your family?”

Sleeping problems: Sleeping problems were measured using three items on a Likert scale of 5 points (from 1 = never to 5 = daily). Items required to indicate how often, in the last 12 months, respondents experienced sleep-related problems (“difficulty falling asleep,” “waking up repeatedly during the sleep,” or “waking up with a feeling of exhaustion and fatigue”).

Work engagement: A three-item version of the Utrecht Work Engagement Scale^55^ measured employees’ work engagement. A 5-point Likert scale was used, from 1 (never) to 5 (always). The items are “At my work, I feel full of energy,” “I am enthusiastic about my job,” and “Time flies when I am working.”

Mental well-being: Mental well-being was measured using the Well-Being Index developed by the World Health Organization in 1998, popularly known as the WHO (5) well-being index. The scale consists of five items on a Likert scale of 6 points, from 1 (at no time) to 6 (all of the time). Samples of items are “Been feeling over the last 2 weeks—I have felt cheerful and in good spirits” and “Been feeling over the last 2 weeks—My daily life has been filled with things that interest me.”

Control variables: The frequency of homework has multiple effects on homeworkers’ well-being.^56,57^ Therefore, we created a dichotomous variable distinguishing the respondents working at home less frequently (grouping together those who responded “several times a month” and “less often,” coded as 1, N_low_ = 5821) or more frequently (grouping together those who responded “several times a week” and “daily,” coded as 2, N_high_ = 5860). Afterward, we tested the direct influence of this variable on the dependent variables of the model.

### Data Analysis

Before the other analyses, an exploratory factor analysis (EFA) was run to check whether each item of the research instrument saturated in the factor theoretically related to it and to carry out a Harman single factor test to check for common method bias.^58^ The EFA was conducted using the maximum likelihood and the Oblimin rotation.

To assess the measurement model and the structural validity of the measures, we ran two confirmatory factor analyses (CFAs), one grouping items in their expected factor and one grouping all the items in a single factor. To assess convergent and divergent validity and the reliability of the scales, we computed, respectively, the average variance extracted (AVE), the maximum shared variance (MSV), and composite reliability (CR). Cronbach alpha was computed for each variable in the study. Descriptive statistics and correlations among variables were then calculated.

Finally, the hypothesized model was investigated using structural equation modeling (SEM). We used the maximum likelihood in the SEM environment to estimate model parameters. We used Fornell and Larcker's^59^ and Hair et al's^60^ indications to evaluate models’ fit and to use appropriate cutoffs. Following Hair et al,^60^ we favored measures such as Root Mean Square Error of Approximation (RMSEA) (cutoff, <0.08) and the incremental measures of Comparative Fit Index (CFI) and Tucker–Lewis Index (TLI) (cutoff, >0.90) over measures such as the *χ*^2^, unreliable in this case because of its high sensitivity to sample size, for evaluating the models’ goodness of fit. We used SPSS 27 and Mplus 8 to perform all analyses.

## RESULTS

### Sample Characteristics

The extraction, from the entire EWCS data set, of the employees engaged in partial or total work-from-home activities resulted in the consideration of 11,501 workers. Participants were, on average, 45.5 years old (SD, 12.9); 48% were female, and 52% were male. Employees working in the private sector were 65.5%, whereas 22.9% reported working in the public sector. The average work hours in a week, intended as the sum of work in the office and at home, was 38.3 (SD, 14.9). Three tenth of the participants (29.1%) worked daily from home; about one fifth of them (20.2%) answered having worked from home several times a week, and the remaining respondents (50.6%) worked from home less frequently. Table 1 summarizes the sociodemographic characteristics of the participants.

**TABLE 1:** Demographic Characteristics of the Research Participants (N = 11,501)

Characteristic	n	%
Age		
Up to 25	614	5.3
26–35	2184	19.0
36–45	3019	26.3
46–55	2973	25.8
56–65	2003	17.4
66 and over	665	5.8
Not reported	43	0.4
Sex		
Men	5975	52.0
Women	5525	48.0
Not reported	1	0.0
Work sector		
Private	7531	66.2
Public	2635	22.9
Joint private-public	380	3.3
Not-for-profit	195	1.7
Other	637	5.5
Not reported	123	1.1
Total hours worked in a week		
Up to 12	839	7.3
13–24	1090	9.5
25–40	5159	44.9
41 and over	3866	33.6
Not reported	547	4.8
Frequency of homeworking		
Several times a month or less	5821	50.6
Several times a week or more	5680	49.4

### Exploratory Factor Analysis, CFAs, Validity, and Reliability of the Scales

The EFA showed no problems with the measurement instruments: the extracted five factors explained 67.05% of the variance, and each one was composed of the expected items with good factor loadings (minimum factor loading, 0.53). Harman single factor test, which forced the extraction of a single factor, demonstrated the absence of common method bias because the extracted single factor explained only 29.37% of the variance. After these preliminary analyses, we continued with the data analysis. Although we decided to test our research model using structural equations, following Hair et al,^60^ we assessed the measurement model through CFAs. In particular, to exclude the absence of a common latent factor and assess the independence of the five measures, we conducted two CFAs, comparing a one-factor model grouping all the study items with a five-factor model in which each item saturated in its expected factor. The results showed that the one-factor model had a very poor fit (*χ*^2^ = 25,401.97; *df* = 104; *P* < 0.001; CFI = 0.56; TLI = 0.50; RMSEA = 0.15; Standardized Root Mean Squared Residual (SRMR) = 0.11). On the other hand, the fit of the five-factor model (*χ*^2^ = 2831.54; *df* = 94; *P* < 0.001; CFI = 0.95; TLI = 0.94; RMSEA = 0.05; SRMR = 0.04) was satisfying, implying structural validity of the model measures. For this model, all items reported saturation values in their factor higher than 0.50.

The minimum AVE score for the five scales was 0.46. Each value was greater than the corresponding MSV score (the highest MSV was 0.35). Furthermore, the square root of each AVE value was higher than the correlations between each considered variable and the other latent constructs, indicating discriminant validity.^59^ All the CR values were over the 0.70 cutoff^60^ and in the range 0.72 to 0.83, suggesting good reliability of the measures. Finally, according to Fornell and Larcker,^59^ although AVE values were slightly lower than the 0.50 cutoff for three of the five study variables (AVE_WFC_ = 0.46, AVE_WENG_ = 0.49, and AVE_W-BEING_ = 0.49), since CR was in every case higher than 0.60 (and 0.70), the convergent validity of the constructs has been considered adequate.

### Cronbach Alphas, Descriptive Statistics, and Correlations Among Variables

Cronbach alphas for the five scales of the model showed values all above the threshold of 0.70, confirming excellent reliability of the model scales again. Together with means, standard deviations, and correlations, such values are reported in Table 2.

**TABLE 2:** Means, Standard Deviation, and Pearson Correlations Among the Study Variables

Variables	Mean	SD	1	2	3	4	5
1. Workload	3.56	1.74	(0.78)				
2. Work-family conflict	2.60	0.90	0.37**	(0.72)			
3. Sleeping problems	2.18	1.00	0.17**	0.35**	(0.79)		
4. Work engagement	4.00	0.69	−0.03*	−0.15**	−0.24**	(0.73)	
5. Mental well-being	4.59	0.86	−0.09**	−0.28**	−0.40**	0.44**	(0.83)
6. Frequency of telework	1.49	0.50	−0.10**	−0.03*	0.02	0.05**	−0.01

N = 11,501.

*P < 0.01.

**P < 0.01.

The average workload reported by homeworkers tended toward high values (mean, 3.56; SD, 1.74), suggesting that homeworkers reported working with moderately tight deadlines and at a high pace. Homeworkers reported having experienced limited level of work-family conflict (mean, 2.60; SD, 0.90) and limited sleeping problems (mean, 2.18; SD, 1.00). On the other side, homeworkers were in many cases engaged with their work (mean, 4.00; SD, 0.67) and in a condition of mental well-being (mean, 4.59; SD, 0.96).

Focusing on the correlations, Table 2 shows that workload was positively correlated with work-family conflict (*r* = 0.37, *P* < 0.001) and sleeping problems (*r* = 0.17, *P* < 0.001), but negatively correlated with mental well-being (*r* = −0.03, *P* = 0.003). Work-family conflict was positively correlated with sleeping problems (*r* = 0.35, *P* < 0.001) and negatively correlated with mental well-being (*r* = −0.28, *P* < 0.001). Sleeping problems had a significant negative association with work engagement (*r* = −0.24, *P* < 0.001) and mental well-being (*r* = −0.40, *P* < 0.001), whereas work engagement had a positive correlation with mental well-being (*r* = 0.44, *P* < 0.001).

### Model Testing

The hypothesized model was tested using SEM. In this model, the control variable of the frequency of homeworking was tested on the mediational variables of work-family conflict and work engagement, since no significant correlations were instead obtained between this control variable and, respectively, sleeping problems and mental well-being.

**FIGURE 2:**
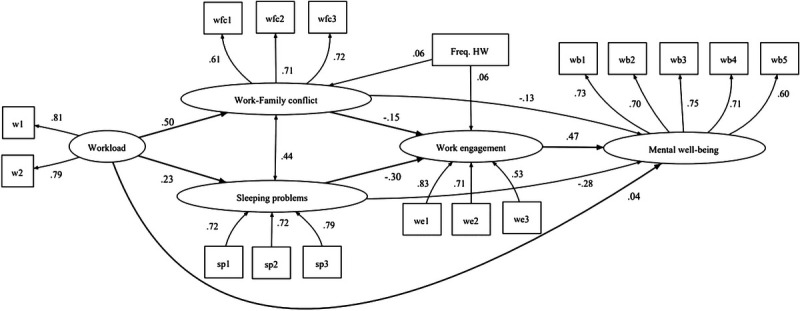
Model standardized results. All the relationships are significant for at least *P* < 0.01.

The model as a whole, with the errors of the variables work-family conflict and sleeping problems correlated to improve the closeness of the model to the reality described by data, reported an adequate fit (*χ*^2^ = 3022.73; *df* = 107; *P* < 0.001; CFI = 0.95; TLI = 0.94; RMSEA = 0.05; SRMR = 0.04). In addition, all the measured items reported saturation values greater than 0.50 in their latent factors, confirming the CFA results and the good validity of the measures. Figure 2 depicts the model results.

According to the model results, the relationship between homeworkers’ workload and mental well-being was small but positive (*β* = 0.04, *P* = 0.001; confidence interval [CI], 0.02 to 0.06). Thus, H1 was not verified, since the hypothesized relationship is significant but, contrary to expectations, positive.

Workload significantly and positively influenced work-family conflict (*β* = 0.50, *P* < 0.001; CI, 0.49 to 0.52; hypotheses H2a supported). In turn, work-family conflict negatively affected work engagement (*β* = −0.15; *P* < 0.001; CI, −0.18 to −0.13) and mental well-being (*β* = −0.13, *P* < 0.001; CI, −0.16 to −0.11). Thus, H2b and H2c were fully supported. Even H2d was supported, and Table 3 shows the indirect effect of homeworkers’ workload on mental well-being via work-family conflict (*β* = −0.07; *P* < 0.001; CI, −0.08 to −0.05).

**TABLE 3:** Indirect Effects of Workload on Mental Well-Being Through the Mediators (H2d, H3d, H4c, H4d, and H4e)

Indirect Effects	*β*
WLD → WFC → MWB	−0.07*
WLD → SP → MWB	−0.06*
WLD → WE → MWB	0.04*
WLD → WFC → WE → MWB	−0.04*
WLD → SP → WE → MWB	−0.03*
Total indirect effect of WLD on MWB	−0.16*

N = 11,501.

MWB, mental well-being; SP, sleeping problems; WE, work engagement; WFC, work-family conflict; WLD, workload.

*P < 0.001.

Regarding the hypotheses about sleeping problems, H3a was supported because homeworkers’ workload was positively related to sleeping problems (*β* = 0.23; *P* < 0.001; CI, 0.21 to 0.25). Sleeping problems was negatively related to work engagement (*β* = −0.30; *P* < 0.001; CI, −0.32 to −0.28) and mental well-being (*β* = −0.28; *P* < 0.001; CI, −0.30 to −0.26), supporting also H3b and H3c. Furthermore, the indirect effect of homeworkers’ workload on mental well-being via sleeping problems was also significant (*β* = −0.06; *P* < 0.001; CI, −0.07 to −0.06), supporting hypothesis H3d (Table 3).

Finally, an unexpected result was observed between homeworkers’ workload and work engagement. Workload was positively, rather than negatively, related to work engagement (*β* = 0.09, *P* < 0.001; CI, 0.07 to 0.11). Hence, hypothesis H4a was not supported, although the relationship is significant and opposite to the hypothesis. However, as expected, homeworkers’ work engagement significantly and positively affected mental well-being (*β* = 0.47, *P* < 0.001; CI, 0.45 to 0.49), supporting hypothesis H4b. Homeworkers’ workload showed also an indirect effect on mental well-being via work engagement (*β* = 0.04; *P* < 0.001; CI, 0.03 to 0.05) (Table 3), supporting hypothesis H4c.

Indirect effects were then observed even in the two serial mediations. The mediations between workload and mental well-being via work-family conflict and work engagement (*β* = −0.04; *P* < 0.001; CI, −0.04 to −0.03), and also that one via sleeping problems and work engagement (*β* = −0.03; *P* < 0.001; CI, −0.04 to −0.03) were significant, thus supporting H4d and H4e.

Finally, the total indirect effect of workload on mental well-being, through the multiple mediators, as shown in Table 3, was negative and significant (*β* = −0.16; *P* < 0.001; CI, −0.17 to −0.14). Hence, the negative indirect effects of workload on mental well-being are higher than the positive direct effect of these two variables; as a result, the total effect of the relationship between workload and mental well-being, calculated as the sum of direct and indirect effects, is therefore negative (*β* = −0.12; *P* < 0.001; CI, −0.14 to −0.10).

Lastly, the control variable of frequency of homeworking revealed significant relationships with the tested variables. Positive, although small, effects were found between frequency of homeworking and, respectively, work-family conflict (*β* = 0.06 *P* < 0.001; CI, 0.05 to 0.08) and work engagement (*β* = 0.06 *P* < 0.001; CI, 0.04 to 0.07).

## DISCUSSION

This study used the COR theory as theoretical background to investigate the relationship between homeworkers’ workload and mental well-being and the mediating effect of work-family conflict, sleeping problems, and work engagement. In light of this approach, we expected that employees’ workload at home was positively related to work-family conflict and sleeping problems and negatively related to work engagement. Furthermore, we expected that work engagement was, in turn, negatively related to work-family conflict and sleeping problems and positively related to mental well-being.

Most of our study hypotheses were supported. Homeworkers’ workload positively affected work-family conflict, sleeping problems, and, surprisingly, work engagement and had a total negative effect on mental well-being.

The positive effect of the workload on work-family conflicts and sleeping problems was also observed in previous studies reporting the positive effect of workload on work-family conflict^30^ and sleeping problems^15,23,61^ in employees working at official sites of their organization. Our result extends findings observed in the official workplace to the field of homework and confirms the applicability of COR theory to homeworking. Investing time and energy resources to cope with an increased workload may result in the depletion of energy resources needed to balance work and family life and have a good quality of sleep, consequently affecting mental well-being resulting from the stress experienced from the loss of resources.

However, study findings also reveal an unexpected result by reporting a positive relationship between workload on work engagement. This unexpected finding, although small (*β* = 0.09; *P* < 0.001; CI, 0.07 to 0.11), is contrary to the one found by Ladyshewsky and Taplin,^62^ who reported that workload negatively affects work engagement. Although this result was unexpected, other studies support the evidence reported in this research, suggesting that workload may not always be harmful but, in some cases, may have a positive effect on work engagement.^43–45,63^ In other words, the workload may not always have a detrimental effect on work engagement. Instead, the relationship between these two variables could be curvilinear in the homeworking context, as already observed in the usual workplace.^45^

Considering that workload was positively related to work-family conflict, sleeping problems, and, at the same time, also positively related to work engagement, our findings support previous studies that identified workload both as a hindrance and a challenge stressor^44,63^ that increases employees’ work engagement to completing their challenging work, while also impacting work-family conflict and sleeping problems that diminish employees’ energy.^43^

Focusing on the relationship between workload and well-being, we point out that, although the direct relationship was small but positive (*β* = 0.04; *P* = 0.001; CI, 0.01 to 05), the total effect of workload on mental well-being, as mentioned above, was instead significant and negative (*β* = −0.12; *P* < 0.001; CI, −0.14 to −0.10), thus suggesting that the three mediators in our model contribute to establishing that too much workload is negative for homeworkers. Therefore, this suggests that intervening in those factors (work-family conflict, work engagement, and sleeping problems) could reduce the negative effect of the workload on homeworkers’ well-being.

The importance of those three mediators is also confirmed by the simple direct relationships they have with mental well-being. This study shows that work-family conflict is negatively related to work engagement and mental well-being, thus supporting prior studies on work engagement^28,32,34^ and employees’ well-being^33,64^ and extending those findings to homeworkers. Although other studies used different theoretical approaches, our results are also coherent with the spiral loss of resources of the COR theory. Sleeping problems experienced by homeworkers had a significant adverse effect on work engagement and well-being, consistently with previous studies conducted in other contexts.^36–39^ Based on the COR theory’s desperation principle, homeworkers may be less inclined to invest more resources into their work task (work engagement) when their self-regulatory resources have not been fully replenished due to sleeping problems.^37^ The loss of this resource, in turn, may explain the loss of the other resource, which is well-being. Thus, our study sheds light on the potential mechanism that the resource loss of time and energy due to high workload compromises sleep quality, leading to the loss of other resources such as well-being.

Finally, despite the frequency of homeworking was marginally related to work-family conflict and work engagement, this variable was not related to mental well-being*. However, we believe that this latter result is also an interesting research finding because it suggests that workers’ mental well-being is not related to the mere frequency of homeworking, but to characteristics of the task and the context in which homeworking is carried out. Nevertheless, we believe these results should be read with caution and also interpreted considering other studies that suggest a curvilinear relationship between frequency of homeworking and some worker satisfaction outcomes.^56,57^

## THEORETICAL AND PRACTICAL IMPLICATIONS

In this study, we contributed to the literature on the relationship between workload and well-being in the context of homework by simultaneously exploring the mediational variables of work-family conflict, sleeping problems, and work engagement.

From a theoretical point of view, since research on the effect of workload on homeworkers’ well-being is limited,^15,16^ we believe our findings, framed in the COR theory,^22^ contribute to homeworking literature by showing that homeworkers’ workload has, on the whole, a negative impact on mental well-being and that workload contributes to increased work-family conflict, sleeping problems, and also work engagement that, in turn, affect mental well-being. This result is coherent with the resource caravans’ principle of the COR theory, which suggests that resources, or threats of resources, do not exist individually but travel in packs.^22^ Thus, workload threatens mental well-being because it affects, at least, other two aspects that can become potential stressors, such as sleep and family relations.

Our results also show that workload is positively related to work engagement and positively related to mental well-being. Considering the second principle of the COR theory, which states that individuals invest resources to protect against resource loss, it seems that employees dedicate time, energy, and mental resources to work (in other words, become more engaged in their work) to compensate the adverse effects of the workload. Hobfoll et al^22^ suggest that individuals, over time, learn how to adapt to stressors and how to use their resources effectively. Thus, a possible explanation of this result is that employees know that workload negatively impacts individual and family resources and, to mitigate such effects, they increase their work engagement to manage their work tasks, complete them quickly and effectively, and dedicate the remaining time to family duties or free time.

On the other side, our study also confirms that workload as a challenging or a hindrance stressor.^43–45^ According to our results, the workload is related to both negative (increased work-family conflict and sleeping problems) and positive outcomes (work engagement), which confirms a complex relationship between workload and employees’ well-being that depends on the mediators included in the studies. Our findings suggest that workload is not only a threatening stressor but also a resource that enhances, through work engagement, employees’ mental well-being. Montani et al^45^ observed that the relationships between workload and work engagement may be curvilinear. Thus, future studies should investigate under which conditions the positive sides of homework workload are observed and how positive and negative effects of workload coexist.

From a practical point of view, this research provides some insights that may help organizations and managers coordinate employees’ work. High amounts of workload are associated with work-family conflict and sleep problems, and these threaten the mental well-being of their employees, potentially affecting their effectiveness at work. On the other hand, we guess that a moderate extent of workload, compared with too low or too high, might enhance employees’ engagement with their work, leading them to feel better and, potentially, work better. Therefore, organizations should pay attention to employees’ workload and identify and avoid to assign tasks, with a too high or low workload to favor employees’ well-being and maximize their efforts.

Our study points out that offering homeworking alone may not be enough. Organizations implementing homeworking should also implement strategies to contain work-family conflict (eg, by considering employees’ childcare needs) and sleeping problems (eg, by promoting proper sleep-wake rhythms, including working on the proper use and correct timing of homework), as well as interventions aimed at fostering work engagement. Such organizational interventions seem promising directions to ensure that workload does not affect the mental well-being of homeworkers.

## LIMITATIONS AND FUTURE RESEARCH

This study has different limitations. In particular, it used a cross-sectional research design, which limits the causal inferences between study variables. In addition, the cross-sectional mediational analysis may show mediational effects that exaggerate indirect effects among study variables that are different from effects observed using longitudinal studies or multiwave design.^65^ To lessen this limitation, we used a large sample size to diminish biases in regression estimates because of measurement errors.^66^ Furthermore, we point out that the study design does not exclude the possibility of reverse mediations between the investigated variables. For these reasons, future research may use a longitudinal design approach to more appropriately support the evidence found here.

Furthermore, another major limitation of the study is that data were collected before the COVID-19 pandemic. Although there are no rational reasons to think about changes in the tested relationships, future studies should verify if, in a postpandemic scenario, the conclusions drawn may still be applicable. Finally, we point out that this study used self-reported measures. Thus, they may lead to exaggeration or understatement on the part of the participants opening up to the tendency of common method bias, which may compromise the study's validity. Therefore, future studies using multirater measures should address this issue.

## CONCLUSION

The present study sheds light on the underlying mechanisms of workload affecting employees’ mental well-being. Findings suggest that the workload experienced by homeworkers is related to work-family conflict, sleeping problems, and work engagement, which, in turn, affect mental well-being. This study contributes to the literature by providing new evidence on the relationship between workload and well-being, offering insights for academic research and organizational interventions on the complex relationship between workload and well-being in homeworkers. We conclude that organizations just offering homeworking without considering needs and duties when working at home are not enough to improve the well-being of homeworkers. Further work on appropriate home working conditions (eg, workload) may represent a good step forward to achieve the purpose of homeworking and improve homeworkers’ well-being. Hence, the present study offered significant knowledge and empirical evidence to help organizational policymakers and managers on the need to pay critical attention to employees’ workload during homeworking.

## Footnotes

* Note: Although not included in our hypotheses, following the suggestion of a reviewer, we tested “frequency of homeworking” using a multigroup approach to highlight potential differences in the model in low- or high-frequency homeworking conditions. The results of this multigroup analysis are not included in this article because they confirmed that all relationships in the research model were significant and, in the same direction, in the low- and high-frequency homeworking conditions. These results are anyway available upon request to the corresponding author.
